# Evaluation of reproductive parameters of vas deferens sperms in Caucasian snake (*Gloydius halys caucasicus*)

**Published:** 2012

**Authors:** Sayedeh Zahra Mozafari, Abdolhossein Shiravi, Fatemeh Todehdehghan

**Affiliations:** 1 *Department of Biology, Islamic Azad University, Damghan branch, Damghan, Iran; *; 2 *Department of Venomous Animals, Razi Vaccine and serum Research Institute, Karaj, Iran.*

**Keywords:** Snake, Sperm, Motility, Vitality, Morphology

## Abstract

Reproductive parameters evaluation is considered as helpful tool for gene bank formation in ecological and economically important animals species. *Gloydius halys caucasicus* is venomous, viviparous pit viper of northwest of Iran. In this research, the spermatic reproductive parameters of this taxon were studied. Twenty six male snakes were collected from Takht-e-Soleiman region between September and October, 2010. Findings revealed that male snakes with body length of 45.07 ± 2.83 cm and body weight of 51.50 ± 10.42 g, and right and left gonads volume of 0.12 ± 0.03 mL and 0.06 ± 0.01 mL are mature ones and sperms concentration in first, middle and final regions of vas deferens duct were, 22.30 ± 19.34 ×10^6 ^mL^-1^, 30.34 ± 11.55 ×10^6^ mL^-1^, and 37.65 ± 16.46×10^6^ mL^-1^, respectively. The sperms motility at three regions of duct were 60.53%, 62.07%, and 60.00% and percentage of immotile sperms in these regions were 39.46%, 37.92%, and 39.84%, respectively. Percentage of morphologically normal sperms was 69.23 ± 10.57% and abnormal sperms was 30.76 ± 10.57%; including 12.69 ± 5.25% spiral tailed, 7.33 ± 4.37% coiled tailed and 4.16 ± 2.51% folded tailed sperms. Percentage of live sperms in the first, middle and final regions of duct were 55.76 ± 10.77%, 58.84 ± 12.77%, and 57.69 ± 9.91%, respectively and percentage of dead sperm in these regions were 44.23 ± 10.77%, 41.15 ± 12.77%, and 42.30 ± 9.91%, respectively. Results suggested, mature sperms with acceptable reproductive quality could be collected from *Gloydiu*s *halys caucasicus* snake of Iran between September and October.

## Introduction

The Caucasian pit viper*, Gloydius halys caucasicus*, is a venomous and viviparous species belonging to the Crotalidae family. It is widely distributed in different parts of Iran^1^^-^^3^ and its habitat is in provinces of Tehran, Gilan, Mazandaran, Golestan, North Khorasan, Razavi Khorasan and Semnan .^1^^,^^2^ Often in temperate zone the mating season of snakes is temporally dissociated from the time of fertilization. Similarly, in males, the mating season is often temporally dissociated from spermatogenesis. In temperate zone pit vipers of North America, estrus, the time when females signal that they are receptive to males, occurs at some time during vitellogenesis. In these pit vipers, vitellogenesis initiates in the late summer or fall. In tropical pit vipers the vitellogenic cycle is continuous (no winter pause) and the mating season occurs at some time during vitellogenesis.^4^ Snakes display considerable variability in reproductive traits among species^5^^,^^6^ exhibiting a wide range of mating systems and male and male reproductive behaviors.^7^^, ^^8^ Saint-Girons has described four patterns of male reproduction.^9^ First, the postnuptial (dissociated) or estival type, which occurs in many temperate and subtropical snake species, is a pattern in which male snake undergo spermatogenesis during the summer, sperms are stored over the winter in the male vasa deferentia and sometimes also in female oviducts (if fall mating occurs), and the principal mating season occurs in spring. This postnuptial pattern of spermatogenesis is predominant among temperate colubrids and crotalines. Second is the prenuptial (associated) or vernal type, which typically occurs in warm climates. In this type, spermatogenesis begins in fall and is completed by the following spring or early summer, when mating occurs. Third is the mixed type characterized by spermatogenesis beginning in spring and being completed one year later, can be one (spring) or two (spring and fall) mating seasons. As with the postnuptial pattern, the mixed type typically occurs in temperate and subtropical species. Finally, the continuous type describes species in environments where there is little seasonal variation in temperature, (e.g., most tropical areas). As the name implies, species that express continuous male reproduction exhibit spermatogenesis and mating behavior throughout the year. Although distinct in definition, these four types of male reproduction in snakes should be viewed as noteworthy points along a cline from distinctly seasonal (estival spermatogenesis) to aseasonal (continuous spermatogenesis), exceptions certainly exist.^9^ Few studies have focused on snake sperm motility^10^^, ^^11^ and all of them have estimated this parameter in a subjective manner. Only one study has thoroughly described the basic sperm dynamic parameters, velocity and motility of a snake species. ^12^ It is widely accepted that the regulation of sperm motility and fertilizing ability depends on the inter action of several factors, among which temperature is of vital importance.^13^ Still, the effect of temperature on sperm dynamic parameters in ectotherms in genera^14^^,^^15^ and reptiles in particular^10^^, ^^16^ remains poorly understood due to the lack of consistent evidence. In a research done in Brazil it was shown that the female* Gloydius halys caucasicus* ovulated in the beginning of spring and mated with male snakes. At the time of ovulation and pregnancy, level of progesterone increased and serum level remained high. Pregnancy lasted for four months, until the end of summer. The average number of neonates was 3 to 9.^17^^, ^^18^ Thus spermatogenesis began from the end of spring in Caucasian snake, and its climax was in the summer, and continued until autumn.^19^ Summer spermatogenesis takes place more often in snake living in temperate and cold regions.^20^ In this research Caucasian viper, (*Gloydius halys caucasicus*)*, *of the Iran was studied. It is among dangerous and poisonous snakes and used for production of polyvalence antitoxin for snake bites. The enzymes existing in its poison are also of the pharmaceutical importance.^21^^, ^^22^

This research contributes to conservation of special and rare species in the country and helps to identify and make optimal use of this reptile in the laboratory research.^21^ More comprehensive scientific information about snakes of Iran could be found in the books: ''Snakes of Iran’’ by Mahmud Latifi,^1^^,^^2^ ''Mar Shenakht'' by Farzanpey^23^ and "Encyclopedia of Hayat- e -Vahsh- e - Iran" by Ibrahimi.^24^ There is no information about spermatic parameters of snakes in Iran as yet. In our research the reproductive parameters of sperm in Iran's Caucasian snake have been studied for the first time.

## Materials and Methods

Twenty six Caucasian male snakes were collected from Takht-e-Soleiman (average annual temperature of 9.5°C, maximum of 27.6 °C, and minimum of -11.3 °C). All procedures were carried out in accordance with ISIRI 7216-2 animal ethical guidelines.^25^ The sperm samples were collected from September to October (in viperidae, vasa deferentia contain sperm from February to October).^26^ The snakes were kept in cages (length 150 cm, width 40-60 cm, height 70 cm) with a temperature of 20-30 °C, 10/14 hr light/dark and fed every second week. Snake was anesthetized, using subcutaneous injection of 1% lidocaine (15 mg kg^-1^, DarouPakhsh, Iran) around the cloacae. The anesthetic was diluted to a total volume of 1.0 mL in normal saline. The total volume of anesthetic was divided between four injection sites (0.25 mL per site) anterior to the cloacae.^11^ Snakes were sacrificed by injection of 1.0 mL ethanol (96%, Bidestan Co., Iran) to each optical cavity. The body length of snakes was measured from the tip of their snout to the vent by ordinary measuring tape.^27^ The testes and vas deferens were removed, then the dimensions of their right and left gonads including lengths, widths and heights were measured by caliper and volumes were calculated by Cha formula.^28^ The vas deferens was divided into three parts of first, middle, and final. Each part was separately placed in Petri dishes, and cut into pieces by sterilized blade and scissors, and immediately kept in vials in 1mL PBS for 45 minutes. During this period the gonads were weighted (Scale No. 11327, Japan). Sperm motility, morphology, vitality, and concentration were studied. The sperm cells were counted using a hemocytometer under 400× and concentration of sperm was calculated according to Rashidi *et al*.^29^ In order to study the sperm motility, four grades were considered: A (quick progressive in straight paths), B (slow progressive in straight or not straight paths), C (motile in place), D (immotile).^30^^-^^32^ Morphology was evaluated by observing 100 sperm cells under microscope at 1000×, and finally the survival period of sperm cells was measured in laboratory temperature of 23 ± 2 °C. 

Statistical analyses were performed by Student *t*-test for comparison of two values at *P *< 0.0001 and one way ANOVA where three values were compared at *P *< 0.05.

## Results

Average weight of the animals, body length, length of tail, details of testes (weight, volume and length) the length of right and left reproductive ducts were measured and have been shown in Table 1. Our results indicated that the right testis was bigger than left one and their average volume, weight and length (Table 1) were significantly different at *P *< 0.0001 and *P* = 0.001, respectively. 

**Table 1:** Some morphometric specifications of *Gloydius halys caucasicus* snake (Mean ± SD).

Variable	Value
Body length (from snout to vent) (cm)	45.07 ± 2.83
Length of tail (cm)	6.07 ± 0.85
Length of left vas deferens from testis to vent (cm)	15.30 ± 1.21
Length of right vas deferens from testis to vent (cm)	11.74 ± 1.003
Weight of right testis (mg)	123.07 ± 16.01
Weight of left testis (mg)	93.07 ± 17.02
Volume of right testis (mL)	0.12 ± 0.03
Volume of left testis (mL)	0.06 ± 0.01
Length of right testis (mm)	21.77 ± 3.70
Length of left testis (mm)	16.69 ± 3.95

Length of left vas deferens duct was longer than right one at *P *< 0.0001. Sperms concentration and vitality in first, middle and final parts of vas deferens, motility, morphology of sperms have been presented in Table 2. Sperm concentrations were not significantly different in three parts of duct (*P* > 0.05), percentages of live and dead sperms were not statistically different in three parts of duct at *P *> 0.05, percentages of motile & immotile sperms (Table 3) differences in three parts of duct were not considered significant (*P *> 0.05).

**Table 2:** Concentration, vitality, survival and morphology of sperms in *Gloydius halys caucasicus *snake (Mean ± SD).

Variable	Value
Sperm concentration in the first region (×10^6 ^mL^-1^)	22.30 ± 19.34
Sperm concentration in the middle region (×10^6 ^mL^-1^)	30.34 ± 11.55
Sperm concentration in the final region (×10^6 ^mL^-1^)	37.65 ± 16.46
Live sperms (first region of duct) (%)	55.76 ± 10.77
Live sperms (middle region of duct) (%)	58.84 ± 12.77
Live sperms (final region of duct) (%)	57.69 ± 9.91
Dead sperms (first region of duct) (%)	44.23 ± 10.77
Dead sperms (middle region of duct) (%)	41.15 ± 12.77
Dead sperms (final region of duct) (%)	42.30 ± 9.91
Sperms survival period (hours)	6.00 ± 2.00
Normal sperms (%)	69.23 ± 10.57
Abnormal sperms (%)	30.76 ± 10.57
Abnormal spiral sperms (%)	12.69 ± 5.25
Abnormal coiled sperms (%)	7.33 ± 4.37
Abnormal short-tailed sperms (%)	8.00 ± 6.99
Abnormal folded tailed sperms (%)	4.16 ± 2.51

**Table 3:** Sperm motility grades in *Gloydius halys caucasicus *snake (ranges of values).

Motile sperm in first region of duct (%)	60.53 (45-84)
Grade A	4.07 (0-13)
Grade B	6.30 (1-13)
Grade C	50.15 (39-67)
Grade D (immotile sperms) (%)	39.46 (16.55)
Motile sperm in middle region of duct (%)	62.07 (43-86)
Grade A	7.46 (0-33)
Grade B	5.53 (3-14)
Grade C	49.07 (38-68)
Grade D (immotile sperms) (%)	37.92 (14-57)
Motile sperm in final region of duct (%)	60.00 (43-75)
Grade A	8.00 (0-23)
Grade B	4.53 (0-10)
Grade C	47.46 (36-66)
Grade D (immotile sperms) (%)	39.84 (25-57)

## Discussion

This study represents basic values of the reproductive potential of Iranian *Gloydius halys caucasicus* which is an important step in the development of assisted reproduction in snakes. The concentration of sperm obtained in our experiment were within ranges of results obtained for checkered garter and *Crotalus durissus terrificus* snakes using similar methods.^33^^-^^35^ Morphologically, 75.70% normal sperm cells reported in corn snakes' ejaculates,^10^ was different from our results (69.23%). The percentage of normal morphology in corn snakes is comparable to that of the ram, bull, boar, stallion, and buck.^36^ In mammals, a small range of morphologic abnormalities are considered normal in a healthy animal. Among the commonly measured semen parameters, morphologic abnormalities have the greatest negative correlation to fertility of farm animals, and heat stress is one of the main causes of sperm abnormalities in these animals. The effect of heat stress on reptile spermatozoa has not yet been assessed. Reptiles are ectotherms and depend on their environmental temperature to regulate their core temperature. It would not be unexpected for a *Gloydius halys caucasicus* snake to experience a 2.7-5.5 °C difference in body temperature in a given day. Although it has not been evaluated, one might expect that semen production would diminish and sperm abnormalities would increase in snakes that experience a large drop in body temperature. Genetics and exogenous drugs have also been associated with sperm cell abnormalities in higher vertebrates. 

In normal fertile individuals up to 50% of sperm cells can have morphological defects (up to 70% according to WHO^31^ criteria and up to 86% according to strict criteria).^35^ Sperm morphology gives information for the function of the reproductive tract and is a predictor of animal's fertility potential. Generally it seems that Caucasian snakes in Iran with SVL 45.07 ± 2.83 cm and testis volume of right 0.12 ± 0.03 mL and left, 0.06 ± 0.01 mL, is mature,^36^ like other snakes in cold and mild regions, have got summer spermatogenesis. ^19^

The findings represent the qualitative and quantitative values of sperms of Iran's Caucasian snake from late September to early October, however in order to obtain higher quality sperms, more work is needed to be carried out.
